# Graphene-enhanced plasmonic nanohole arrays for environmental sensing in aqueous samples

**DOI:** 10.3762/bjnano.7.150

**Published:** 2016-11-01

**Authors:** Christa Genslein, Peter Hausler, Eva-Maria Kirchner, Rudolf Bierl, Antje J Baeumner, Thomas Hirsch

**Affiliations:** 1 Institute of Analytical Chemistry, Chemo and Biosensors, University of Regensburg, 93040 Regensburg, Germany; 2 Sensorik-ApplikationsZentrum, OTH Regensburg, Franz-Mayer-Str. 1, 93053 Regensburg, Germany

**Keywords:** diethyl phthalate, environmental sensing, nanohole array, nanosphere lithography, surface plasmon resonance

## Abstract

The label-free nature of surface plasmon resonance techniques (SPR) enables a fast, specific, and sensitive analysis of molecular interactions. However, detection of highly diluted concentrations and small molecules is still challenging. It is shown here that in contrast to continuous gold films, gold nanohole arrays can significantly improve the performance of SPR devices in angle-dependent measurement mode, as a signal amplification arises from localized surface plasmons at the nanostructures. This leads consequently to an increased sensing capability of molecules bound to the nanohole array surface. Furthermore, a reduced graphene oxide (rGO) sensor surface was layered over the nanohole array. Reduced graphene oxide is a 2D nanomaterial consisting of sp^2^-hybridized carbon atoms and is an attractive receptor surface for SPR as it omits any bulk phase and therefore allows fast response times. In fact, it was found that nanohole arrays demonstrated a higher shift in the resonance angle of 250–380% compared to a continuous gold film. At the same time the nanohole array structure as characterized by its diameter-to-periodicity ratio had minimal influence on the binding capacity of the sensor surface. As a simple and environmentally highly relevant model, binding of the plasticizer diethyl phthalate (DEP) via π-stacking was monitored on the rGO gold nanohole array realizing a limit of detection of as low as 20 nM. The concentration-dependent signal change was studied with the best performing rGO-modified nanohole arrays. Compared to continuous gold films a diameter-to-periodicity ratio (*D*/*P*) of 0.43 lead to a 12-fold signal enhancement. Finally, the effect of environmental waters on the sensor was evaluated using samples from sea, lake and river waters spiked with analytically relevant amounts of DEP during which significant changes in the SPR signal are observed. It is expected that this concept can be successfully transferred to enhance the sensitivity in SPR sensors.

## Introduction

Plasticizers are additives used in plastic industry, personal care products and especially in polyvinyl chloride (PVC) products. The most common plasticizers are phthalate acid esters (PAEs) [1]. Since PAEs are not chemically bound to the polymeric matrix, they can leach into the environment. The resulting wide distribution in aqueous systems, such as lakes and rivers, and disturbances of the ecological environment are caused by accumulation of PAEs in natural waters [2–3]. It has been reported that PAEs trigger adverse effects on human health and are readily absorbed through the skin. They can cause feminization of male infants, impact genital development and testes maturation. Metabolic products are also potential thyroid hormone disruptors [4–6]. Because of their carcinogenic and toxic characteristics determination of PAEs in environmental water is an urgent task. Most widely used techniques are gas chromatography and high performance liquid chromatography coupled with mass spectrometry (GC–MS and HPLC–MS), however often enrichment and extraction steps prior to the analysis are necessary [7]. An online detection system for natural water with detection limits in the environmental interesting concentration is important for water safety and direly needed.

Surface plasmon resonance spectroscopy (SPR) is a widely-used technique for quantifying and characterizing biomolecular interactions in biosensors for medical diagnostics, food safety and environmental monitoring providing important features such as real time measurements, high sensitivity and label-free assay [8]. The detection of highly diluted concentrations and small molecules (<200 Da) remains challenging within SPR sensing [9]. For (bio)analytical applications the sensitivity needs to be enhanced to achieve low detection limits. To address this issue nanomaterials ranging from metallic nanoparticles, carbon-based structures to liposomes were used [10–12]. Plasmonic transducers are sensitive to changes of optical properties such as the dielectric constant and hence the refractive index next to their surface. The exponential decay of the plasmonic field generates a response affected by the penetrated volume within the solution [13]. Within conventional SPR sensing propagating surface plasmons (PSP) are the main parameter, defined as propagating charge oscillations on the surface of a thin metal film. At a visible wavelength the decay of PSP on a planar surface is approximately half of the excitation wavelength and in the range of a few hundred nanometers [14]. For localized surface plasmons (LSP) occurring at nanostructures, the values are significantly smaller and are in the range of 5–60 nm [14–15]. An enhancement of local electromagnetic fields and intense absorption bands due to excitations of electrons at the nano-structures, results in a high sensitivity towards local changes of the refractive index [16]. A variety of nanostructured substrates, such as nanostructured arrays, has been designed and applied to bioanalytical sensing applications [17–20]. Nanohole arrays, which are characterized by combining localized and propagating surface plasmons, offer a possibility to tune the plasmonic features and therefore optimize the sensing performance for a specific application [21]. They have been shown to provide better sensitivity in wavelength dependent SPR sensing. However, most commercial SPR devices are based on angle scanning by illumination at a constant wavelength and no studies are available investigating this interesting plasmonic effect [17,22].

Nanohole arrays have been first fabricated 1995 by Masuda and Fukuda using a replication process of an anodized alumina structure [23]. Since then, a vast number of techniques has been invented. For example, as focused ion beam (FBI) milling allows a control of the size and shape of the nanoholes with good reproducibility it has been applied for biosensor development and theoretical studies. With high fabrication costs and long milling times it is not adaptable to large volume manufacturing [24–26]. Standard lithography techniques can instead be used such as soft embossing. An imprinting mask is prepared by e-beam lithography and by printing numerous times on a surface, large areas of nanoholes are created [27–30]. Since for each different nanohole layout a new mask needs to be fabricated, this method is still time consuming and unfavorable for optimization studies. To provide tunability, rapid fabrication and low manufacturing cost that can also easily be done in low-class clean room areas, techniques such as polymer blend lithography or a modified nanosphere lithography (NSL) technique were recently developed [31–32]. Using colloidal lithography disordered nanoholes can be obtained. A combination of NSL with electrochemical deposition, ion-polishing, plasma treatment and glancing-angle deposition produces ordered nanohole arrays. Therefore, NSL is a promising tool to produce nanostructured substrates.

**Figure 1:**
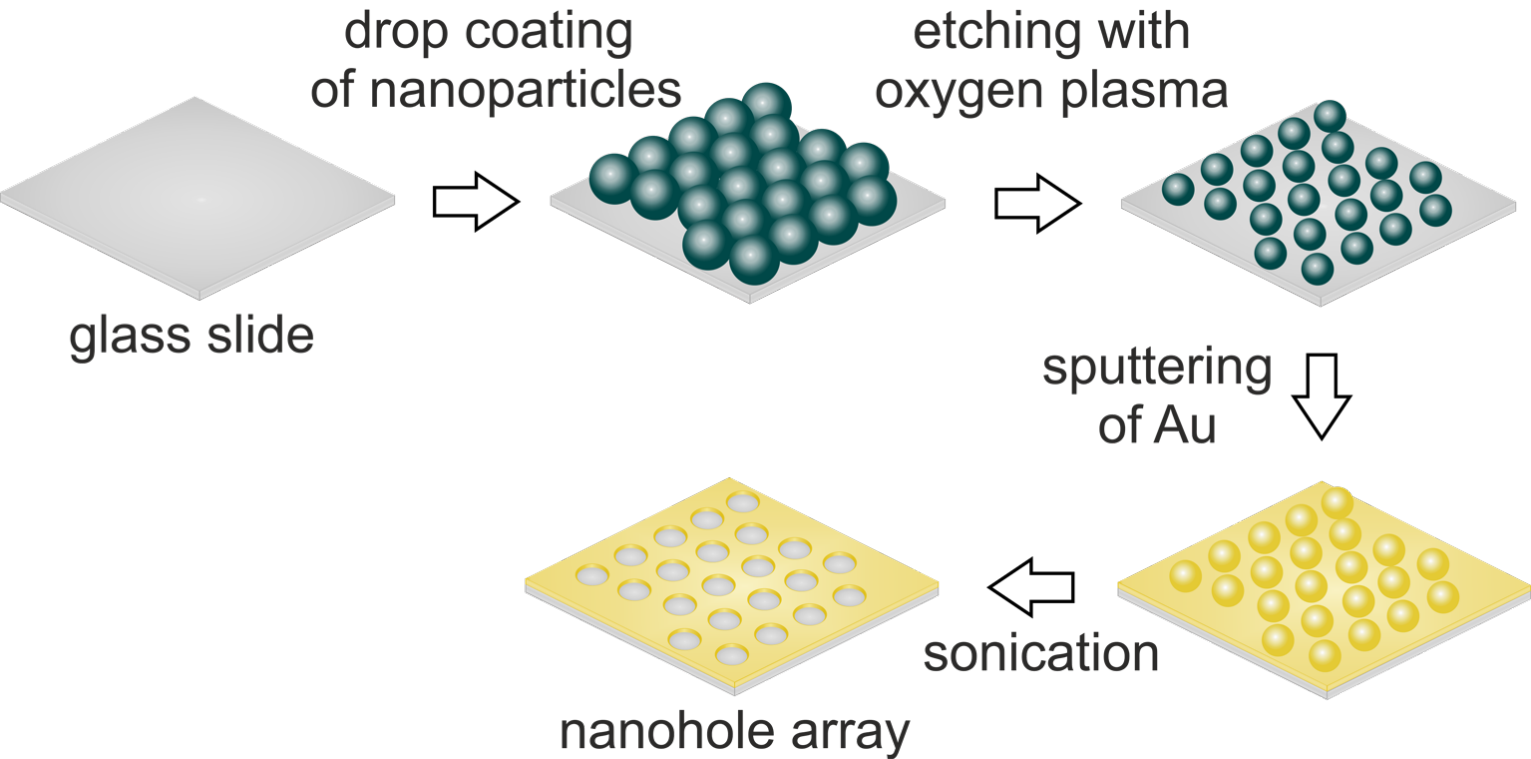
Outline of the fabrication steps to form a nanohole array with a modified nanosphere lithography technique.

Here, nanohole arrays were prepared by a modified nanosphere lithography (Figure 1). The dominating parameters for sphere mask formation are the evaporation rate and the particle content. Both can be tuned very precisely [33]. The most characteristic parameter for nanohole arrays is the diameter-to-periodicity ratio (*D*/*P*), as visualized below in Figure 4B. Hole diameter (*D*) and periodicity (*P*, distance between the centres of neighbouring holes) both significantly affect the plasmonic properties and therefore the sensitivity of nanohole arrays [34].

For an analytical application the gold layer needs to be modified with a receptor layer. Reduced graphene oxide (rGO) is a very interesting receptor layer as it serves two purposes. On the one hand, it improves the sensing performance as its high surface-to-volume ratio leads to more efficient adsorption of molecules together with local plasmonic enhancement effects [35–36]. Thus, systems consisting of a plasmonic nanostructure and graphene are referred to as plasmon–graphene hybrids [37]. On the other hand, interactions of molecules with aromatic systems via π-stacking is strongly promoted by the sp^2^-hybridized carbon atoms arranged in a honeycomb structure. In this study a sensor for diethyl phthalate as model analyte was developed. It is known that this type of plasticizers adsorbs on polystyrene resins by multiple adsorbent–adsorbate interactions such as hydrogen bonding and π-stacking [38], which makes them an ideal analyte for the evaluation of the graphene-modified gold surfaces in SPR.

Nanostructured surfaces are promising in enhancing the signals in surface-sensitive techniques. The excitation of localized surface plasmons are known to improve Raman signals on structured metal surfaces significantly, and often utilized in sensing systems. A Web of Science survey revealed more than 1600 publications on the concept of surface-enhanced Raman scattering (SERS) in the year 2015 alone. In contrast, in the same year only 25 publications report on the enhancement of SPR signals by introducing nanostructured surfaces. One reason can be attributed to the different size of the sensing spots used in these two prominent techniques. Commercial SPR devices usually illuminate spots in the range of several square millimetres. This is about 3·10^7^-times larger than the area in Raman microscopy using an 100× objective. This comes with the need to fabricate regular nanostructures in large lateral dimensions, while also ideally delivering fast and reproducible substrates. These requirements are easily met in nanosphere lithography as sphere size and monolayer formation allow for facile nanohole array design and systematic variation of its properties.

## Results and Discussion

**Figure 2:**
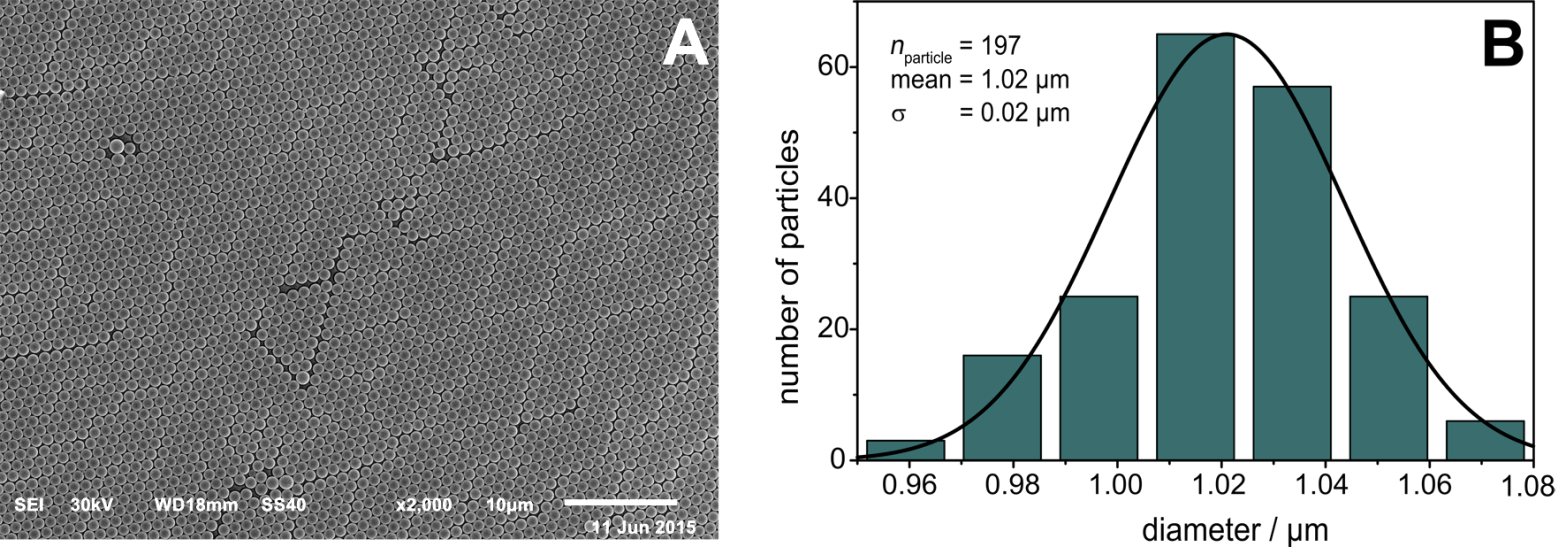
SEM image (A) of a densely packed monolayer of polystyrene particles with a diameter of 1.02 μm. Substrates were covered by ~45 nm Au with a ~3 nm Ti adhesion layer. Scale bar is 10 μm. (B) The respective particle size distribution fitted with a Gaussian function.

A nanohole array modified SPR chip was fabricated according to the method described by Masson et al. [39–40]. Drop casting of the polystyrene particles on a clean glass slide leads to a densely packed monolayer on the substrate (Figure 2). The sphere mask consists of ordered areas of several square millimetres covering more than the optical spot size of the SPR device (0.23 cm^2^). Highly ordered monolayers are mandatory to obtain a periodic and defined structuring of the substrates, as described for signal enhancement in wavelength-dependent SPR studies [41].

The diameter and periodicity of the nanoholes strongly influences the plasmonic excitation and the sensitivity [42]. With hole sizes smaller than the wavelength of incident light, a large variety of optical properties such as filtering of wavelength and enhanced transmission of light through the holes occurs [43]. Understanding the principles of the optical properties of the arrays with a hole diameter smaller than the wavelength of light has been in the focus of research in the last years [44–45]. In one example, the influence of the nanohole diameter at a fixed periodicity on the transmission spectra was investigated. With decreasing hole diameter the SPR wavelength shifts to shorter wavelengths and hence changes the optical properties [46]. Yet, much is still unknown and further understanding of the potential in sensing applications of substrates with both surface plasmon modes can be achieved by comparison of their analytical properties. Thus different diameter-to-periodicity ratios (*D*/*P*) for a specific analytical application were studied, as the optimal plasmonic properties, e.g., penetration depth of the plasmonic field and sensitivity depend on the excitation method [41].

**Figure 3:**
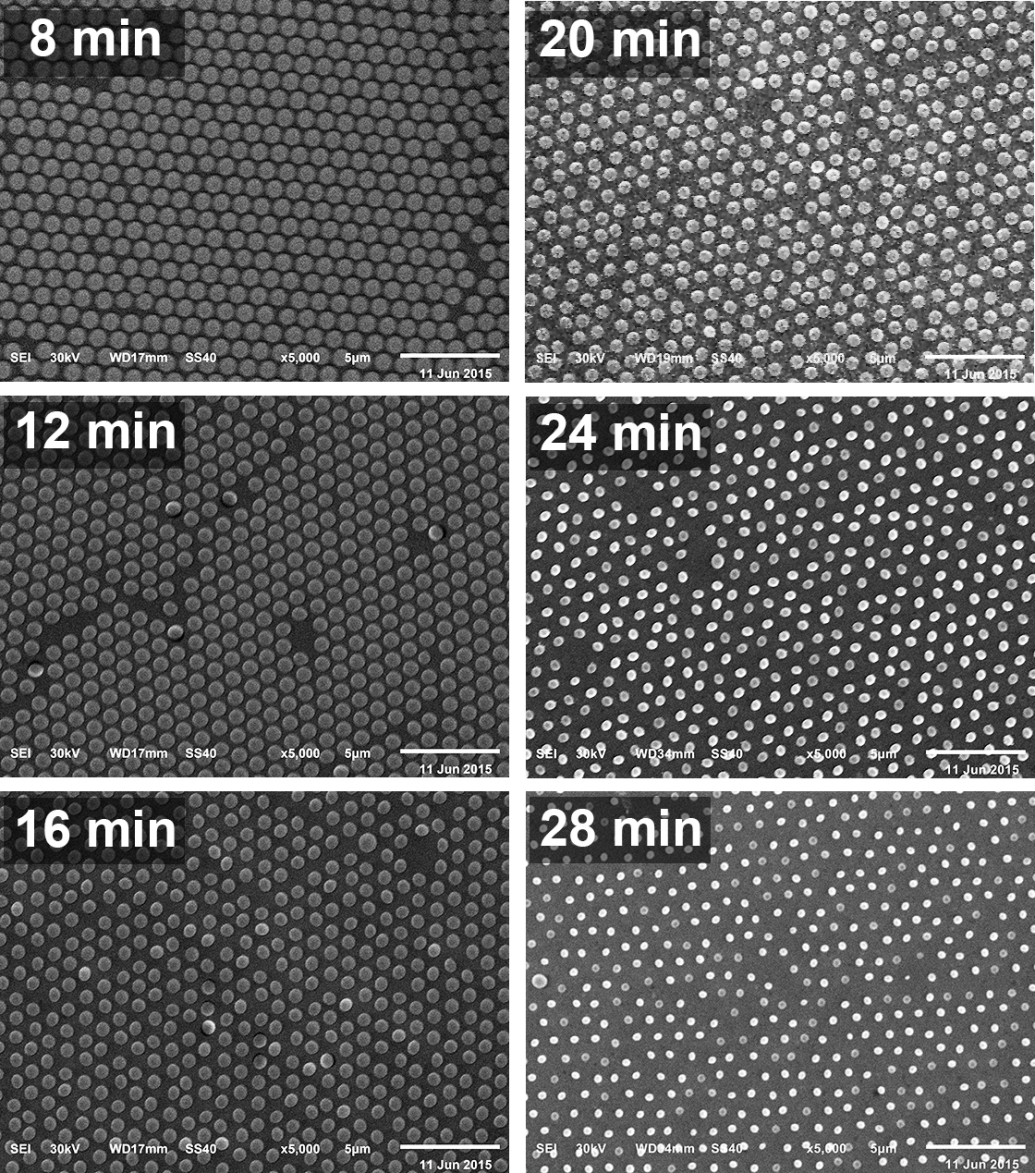
SEM images of the sphere masks etched by oxygen plasma at 18 W with different times (8–28 min). A decrease in the diameter of the polystyrene particles with an increase of the etching time can be seen. The periodicity is not affected by the etching process as the spheres remain at their initial position. Substrates were covered by ~45 nm Au with a ~3 nm Ti adhesion layer after the etching process. All scale bars are 5 μm.

In order to vary the *D*/*P* (Figure 3) of the nanostructured substrate, the spheres were changed in size without altering their position on top of the glass slide by plasma etching. The periodicity (*P*) is not affected by this process, as the particles remain at their initial positions. Spheres were etched from 0.82 to 0.36 µm with a small standard deviation of a maximum of ±0.05 µm (particle-size distribution shown in Figure S1, Supporting Information File 1). The hexagonal arrangement of the closed-packed monolayer is still visible after oxygen plasma treatment. By varying the etching time, a linear relationship to the particle diameter was determined (Figure S2, Supporting Information File 1). Hence, desirable hole diameters and consequently desirable *D*/*P* can be fabricated easily by adjusting the etching time.

**Figure 4:**
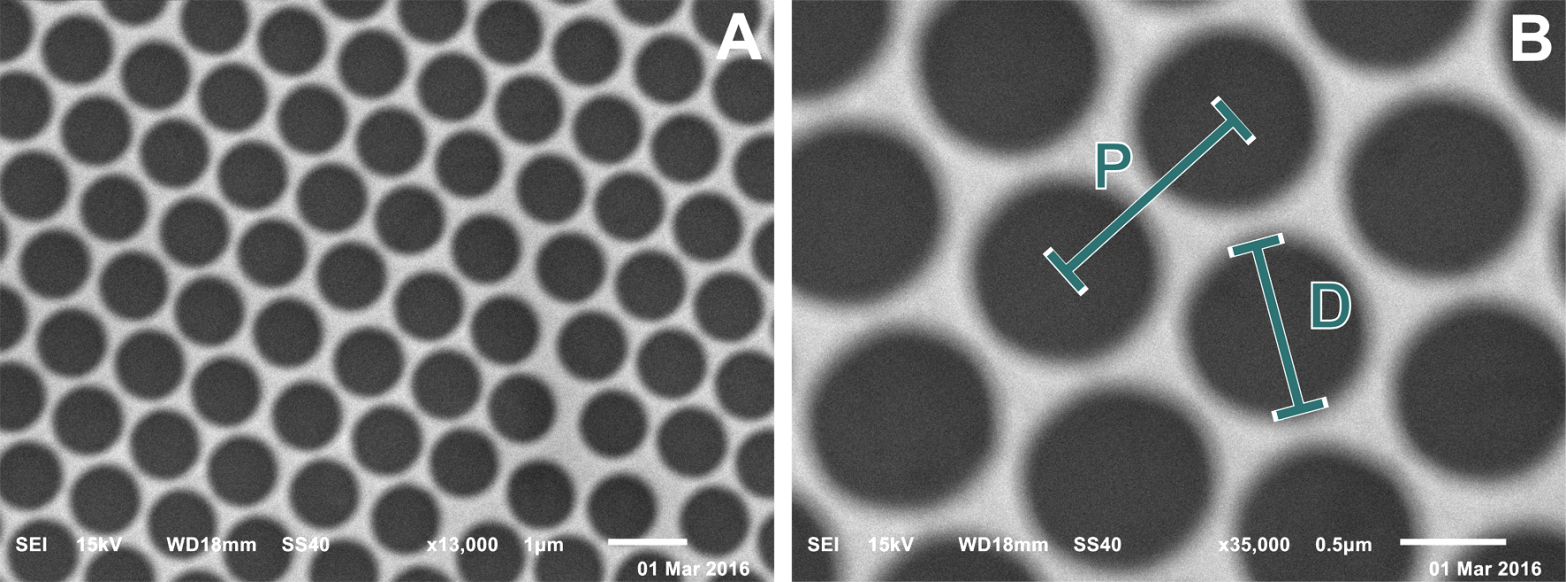
SEM image of a nanohole array with a hole diameter of 0.82 ± 0.04 µm. Spheres were etched for 8 min. Substrates were covered by ~45 nm Au with a ~3 nm Ti adhesion layer. Particles were removed by sonication in ethanol. Scale bars are 1 μm (A) and 0.5 µm (B).

The sphere mask was then covered by a thin film of gold with a thickness of ~45 nm. This thickness is identically to commercial SPR slides with a continuous gold film and was chosen for providing optimal results in angle-dependent SPR devices using 650 nm excitation [47–48]. In a final step the substrates were sonicated in ethanol to remove the PS spheres. Figure 4 shows an SEM image of a glass chip covered by the thin gold film structured as a nanohole array with a *D*/*P* ratio of 0.80, high regularity and sharp borders.

**Figure 5:**
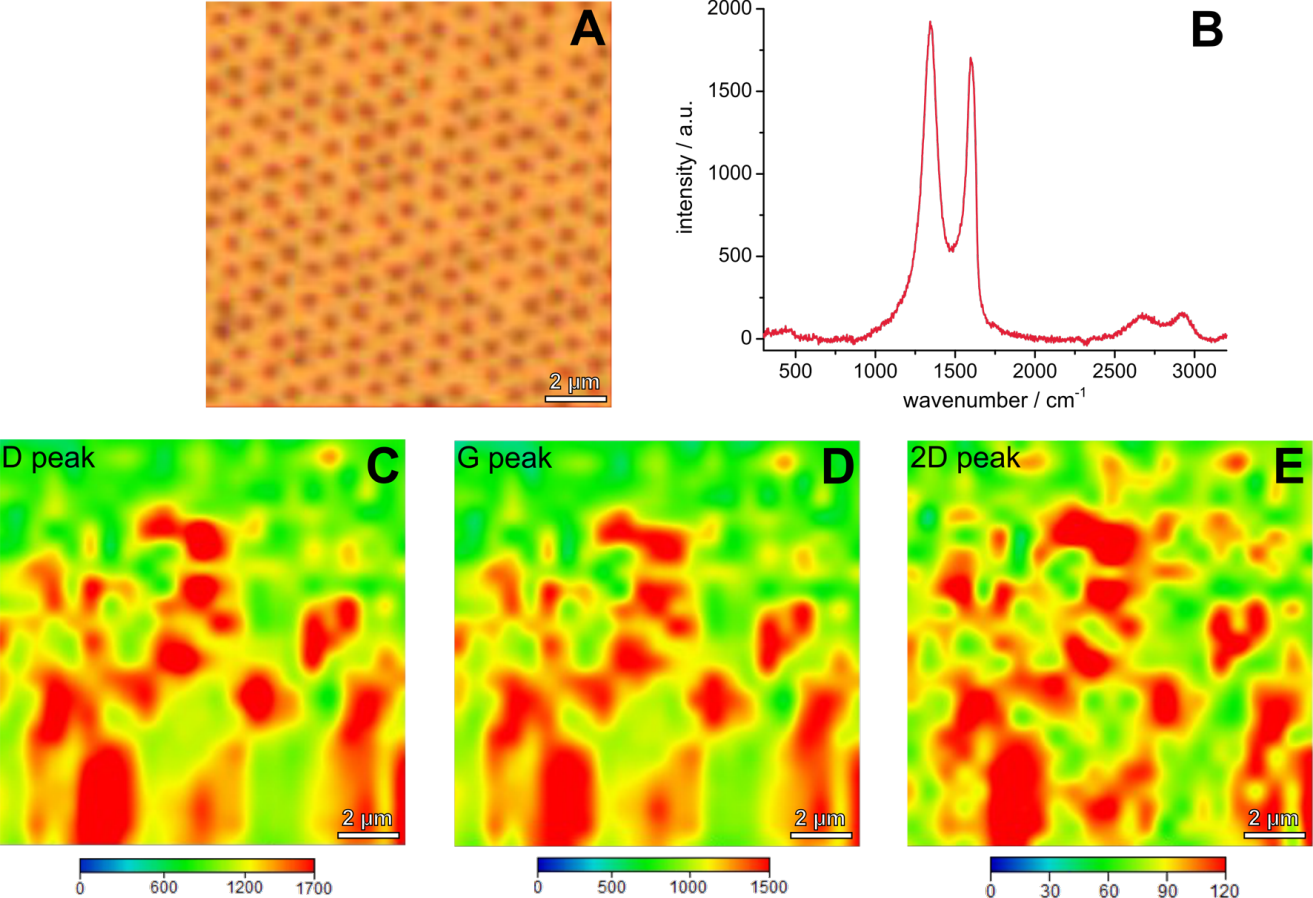
Microscopic image (A), exemplary Raman spectrum (B) and Raman maps (C–E) of the sensor slide consisting of rGO on a nanohole array with a *D*/*P* ratio of 0.43. The maps show the Raman intensity of the D-peak at 1345 cm^−1^ (C), the G-peak at 1603 cm^−1^ (D) and the 2D-peak at 2682 cm^−1^ (E) on the area shown in the microscopic image (A).

For the fabrication of the plasmon–graphene hybrids, the nanostructured substrates were functionalized with rGO via spin coating. The resulting two-dimensional graphene nanomaterial was characterized using Raman microscopy (Figure 5).

Reduced graphene oxide is identified by the three distinct Raman bands at 1345 cm^−1^ (D-Peak), 1603 cm^−1^ (G-Peak), and 2682 cm^−1^ (2D-Peak) (Figure 5B) [49]. The presence of multilayers is indicated by the low intensity of the 2D-peak at 2690 cm^−1^ [50]. Raman maps showing the intensity of the D-, G- and the 2D-peaks over an area of 13 × 13 µm demonstrate a full coverage of the nanohole array with rGO. Deviations in the Raman intensity can be ascribed to inhomogeneous multilayers, which is due to the spin coating process.

The presence of localized surface plasmons was demonstrated by analysing the resonance curves before and after functionalization with rGO (Table 1). Normally, it is expected that a decrease of the amount of gold is accompanied by a decrease in the sensitivity. However, the results display a contrary trend, which in turn indicates the excitation of localized surface plasmons arising from the nanostructures on the substrate and interactions with the rGO [51]. In fact, it was found that, in general, all nanohole arrays regardless of their *D*/*P* ratios demonstrated a higher shift in the resonance angle of 250–380% compared to a continuous gold film. Somewhat surprisingly the improvement in surface sensitivity achieved for different *D*/*P* ratios of 0.35 to 0.58 is almost of the same order. The SPR response is affected by the presence of different plasmonic properties and accordingly strongly influenced by the dimensions of the nanostructures. A possible explanation for the findings are variations in the plasmonic band structure, leading to different excitation wavelengths and penetration depths. It has been reported that the periodicity only slightly impacts the shape of curve for smaller *D*/*P* ratios (<0.5) and is similar (low values for the full width at half maximum (FWHM)) to a continuous gold film, whereas for larger *D*/*P* ratios (above 0.5) the curve is broadened [52]. Here, i.e., for wavelength-dependent SPR, the shape of the SPR curve was investigated as a function of the *D*/*P* ratio. It was found that nanohole arrays with *D*/*P* ratios of 0.35 and 0.43 display FWHM values of 3.5° and 4.0°, which are similar to that of a continuous gold film with 3.5°. Starting with a *D*/*P* ratio of 0.58 the FWHM is increasing (4.9°). For higher *D*/*P* ratio values from 0.63 to 0.80 high FWHM values of 5.9° to 6.4° are observed. As a less steep rise of the curve results in a lower sensitivity and as with decreasing *D*/*P* ratio a sharper curve and hence a higher sensitivity can be observed, the three *D*/*P* ratios of 0.58, 0.43 and 0.35 were chosen for additional studies.

**Table 1:** Change in SPR resonance angle of nanohole arrays with different *D*/*P*-ratios compared to a continuous gold film. Each value represents the average value of three measurements. Errors indicate the standard deviation of these measurements.

	continuous film	*D*/*P* of the nanohole array					
		0.80	0.73	0.63	0.58	0.43	0.35

Δθ_SPR_ / °	0.13 ± 0.08	0.5 ± 0.1	0.49 ± 0.03	0.6 ± 0.1	0.6 ± 0.2	0.5 ± 0.1	0.6 ± 0.2

To demonstrate the advantage of the plasmon–graphene hybrids within its sensing properties, the detection of diethyl phthalate as a plasticizer in water was investigated. The concentration-dependent signal change was studied with the best performing rGO-modified nanohole arrays, and compared to rGO-modified continuous gold films (Table 2). The binding of the analyte to rGO was studied by SPR measurements resulting in a saturation curve in good accordance to the Langmuir model (Equation 1):

[1]
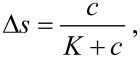


where Δ*s* is the signal change, *c* is the DEP concentration and *K* represents the equilibrium dissociation constant.

**Table 2:** Binding constants *K*_A_ for rGO-modified nanohole arrays with *D*/*P* ratios 0.58, 0.43 and 0.35 compared to a continuous rGO-modified gold film. Data were fitted with the Langmuir equation (Equation 1). Fitting parameters are shown in Table S3 (Supporting Information File 1).

	continuous gold film	*D*/*P* of the nanohole array		
		0.35	0.43	0.58

*K*_A_ / 10^6^·M^−1^	6 ± 1	5 ± 1	7 ± 0.9	5 ± 1

All binding constants are almost identical, with the highest value for a *D*/*P* ratio of 0.43. This indicates a reproducible rGO layer deposition and no influence of the nanohole array structure on the interaction of DEP with rGO via π-stacking.

**Figure 6:**
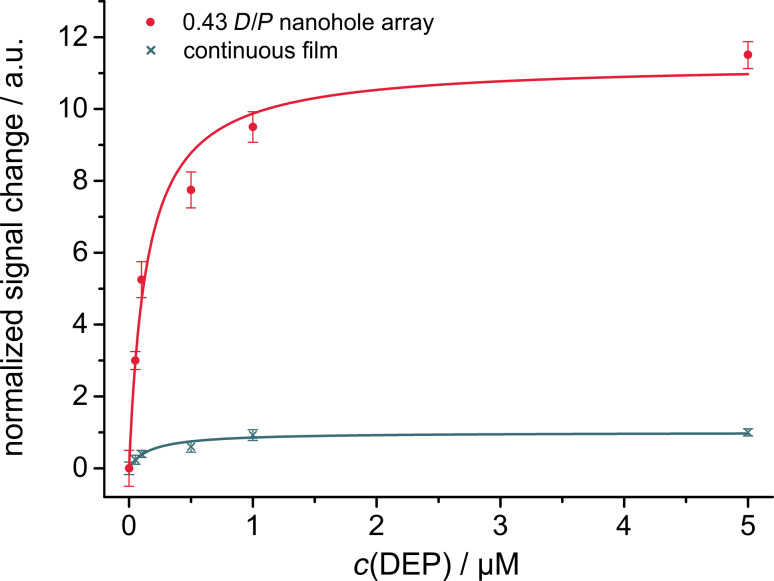
Normalized signal change at a constant angle in response to binding of DEP to rGO-modified nanohole arrays (*D*/*P* = 0.43) covering a concentration range from 0.05 to 5 µM. For comparison the response of a continuous gold film modified with rGO is shown. Error bars indicate S/N. Data were fitted with the Langmuir equation (Equation 1).

Based on these findings a nanohole array with a *D*/*P* ratio of 0.43 was applied as sensing substrate for the analysis of DEP in double distilled water. The signal change of this system with increasing DEP concentrations was determined and compared to a continuous gold film (Figure 6).

For the nanohole array with a *D*/*P* of 0.43, saturation is almost reached at 5 µM (97% according to the Langmuir fit) and a roughly 12-fold enhancement of the maximum signal response compared to a continuous gold film is observed. Therefore, a 10-times better limit of detection (LOD) of ca. 20 nM is found when measuring with substrates with *D*/*P* = 0.43 compared to a continuous gold film (ca. 190 nM). The concentration covers the guideline values of the World Health Organization in fresh and drinking water for bis(2-ethylhexyl)phthalate (3–40 nM), the most widespread phthalate commonly used as reference for other phthalates [53–54]. The substrate provides therefore a very promising platform for detecting DEP in real water samples at low concentrations.

**Figure 7:**
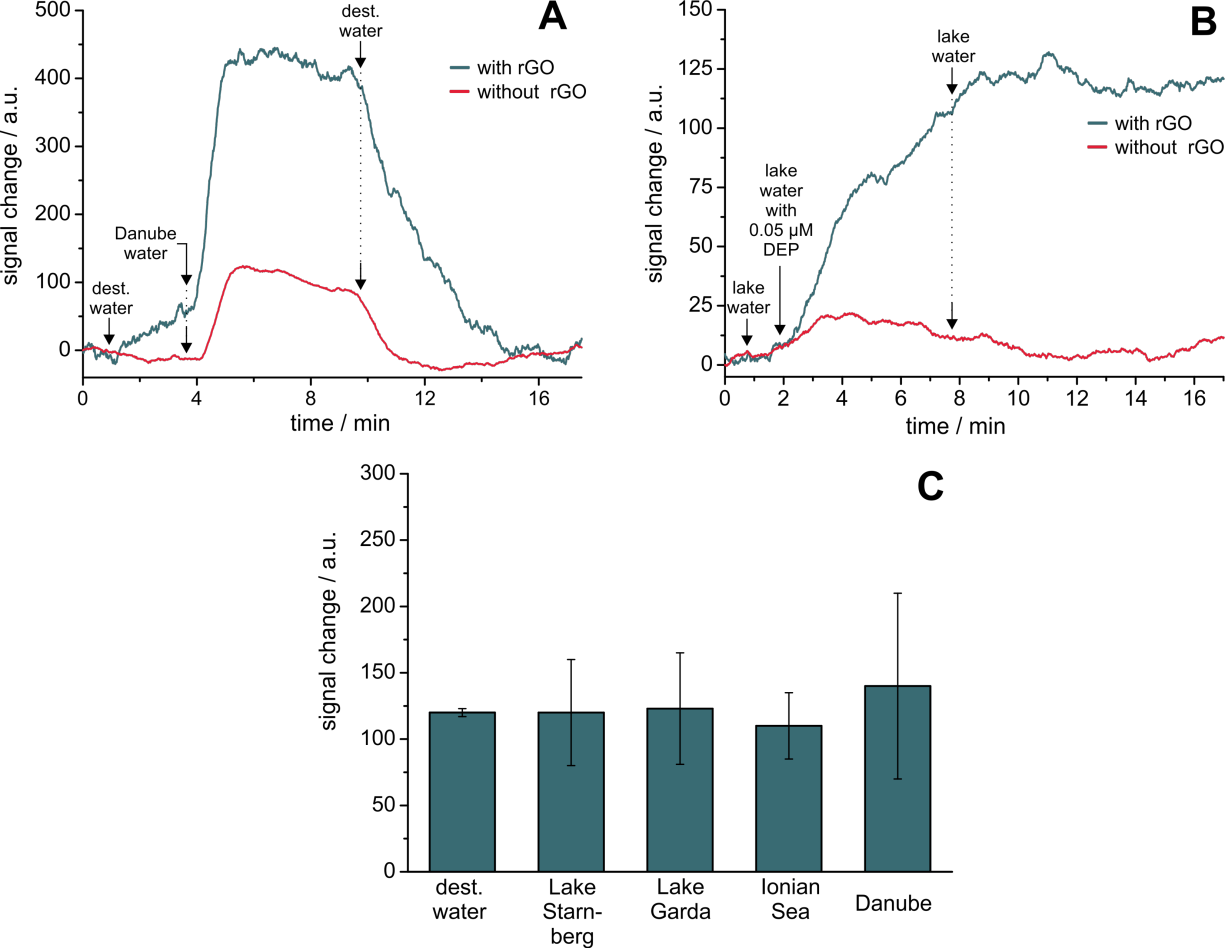
Exemplary signal traces at a constant angle for a nanohole array with *D*/*P* = 0.43 (A) with and without rGO receptor layers recorded for the addition of Danube water and recovery with the addition of distilled water, and (B) of Lake Starnberg water followed by the same water spiked with 0.05 µM DEP and subsequent washing with original Lake Starnberg water again. (C) Signal change for various water samples for a nanohole array (*D*/*P* = 0.43) with rGO. Samples were spiked with 0.05 µM DEP.

The applicability of plasmon–graphene hybrids in commercial SPR devices measuring the angle dependence at a constant wavelength is demonstrated by the investigation of real water samples. For all real water samples higher signal changes were achieved when the nanohole arrays were functionalized with graphene shown exemplary with water from the river Danube (Figure 7A). By switching back to washing conditions, the original baseline was again obtained. That means that no specific binding between sample components and the graphene layer was formed. Thus, synergistic plasmonic effects caused by the interplay of the localized surface plasmons with the plasmonics of the overlaid carbon nanostructures lead to the significant signal enhancement. Secondly, when the water samples were spiked with the model analyte DEP, which will bind to graphene via π-stacking, the binding was stable even upon washing with the water sample without spiked DEP (Figure 7B). Extensive washing with double distilled water for several hours is needed to recover the sensor surface again (data not shown). Finally, two lake water (Lake Starnberg, Germany and Lake Garda, Italy), a sea water (Ionian Sea, Greece) and a river water (Danube, Germany) samples were spiked with 0.05 µM DEP. The obtained signal changes were compared to 0.05 µM DEP in double distilled water. In all cases this low concentration of the analyte was recovered with a satisfying yield (Figure 7C). From these results one can conclude that SPR on nanohole array modified with rGO enables a label-free online system to monitor changes in concentrations of phthalates as an example for analytes with the ability of π-stacking. This clearly demonstrates the advantage of the interplay of the nanostructured gold layer with the carbon nanomaterial as neither rGO on continuous gold nor a nanohole array without rGO modification will lead to such sensitive signal changes.

## Conclusion

Nanosphere lithography was demonstrated to be a versatile technique for the fabrication of size-tailored nanohole arrays on a large scale for plasmonic enhancement in angular-dependent surface plasmon resonance with a constant wavelength setting. Plasmon–graphene hybrids were fabricated by spin-coating of the carbon nanomaterial on top of the substrates. This system was able to demonstrate a 10-fold lower limit of detection for small molecules than continuous gold films in a surface plasmon resonance affinity set-up. At the same time, very similar binding constants between the continuous gold film and various nanohole arrays emphasize that the nanostructured surface does not affect the interaction of DEP with rGO. The feasibility of the signal enhancement by localized plasmons was demonstrated for the detection of small molecules such as DEP in environmental water samples without pre-treatment. This enables the detection of even small molecules at low concentrations. Nevertheless, selectivity still needs to be improved. Specific receptors can be attached to the carbon nanomaterial or selective filters based on molecular imprinted polymer films can be applied. The combination of several semi-specific sensors to an artificial nose with chemometric analysis of a complex matrix will also offer a possible solution. Therefore, it is expected that hybrid materials consisting of nanostructured gold together with two-dimensional nanomaterials will be attractive in designing new sensor applications based on SPR transduction.

## Experimental

### Nanohole array fabrication

All substrates are based on glass slides (20 × 20 mm^2^) of F1-Type with a refractive index of 1.61 (Mivitec GmbH, Sinzing, Germany). All glass slides were cleaned in piranha solution for 90 min and in a mixture of water, ammonia and hydrogen peroxide at a 5:1:1 (v/v/v) ratio for 60 min in an ultrasonication bath. Between treatments the glass slides were rinsed with water and sonicated three times in water for 15 min. Each time the water was exchanged.

The fabrication of nanohole arrays consists of several steps [39–40]. First a sphere mask of a hexagonal, closed packed, two dimensional crystal of polymer particles needs to be formed via self-assembly by a slow evaporation process. Subsequent etching of the particles creates a void between neighbouring particles, generating a non-close packed ordered sphere monolayer. The obtained sphere mask acts as a pattern during gold deposition. Varying the etching time results in different diameters of the spheres and respectively holes. Lift-off of the sphere mask is achieved by sonication in ethanol.

The sphere mask is gained by drop-coating of 40 µL of a water/ethanol solution 87:13 (v/v) containing 13 mg·mL^−1^ polystyrene particles on a clean and dry glass slide. The polystyrene particles have a diameter of 1.04 μm (SD = 0.04 μm, microparticles GmbH). Covering with a Petri dish allows a slow evaporation rate, resulting in a close-packed monolayer. The sphere masks were dried overnight. In order to create a nanohole array the diameter of the spheres need to be etched by reactive ion etching using oxygen plasma (Plasmalab 80 Plus, Oxford Instruments, Abingdon, United Kingdom) prior to metallization. Different diameters of the polystyrene spheres were achieved by varying the etching time from 8 to 28 min at 18 W. On the etched sphere mask a thin layer of ca. 3 nm Ti was deposited before Au deposition (ca. 45 nm). The resulting arrays were analyzed using SEM. Finally, the PS spheres were removed from the surface by sonication in ethanol for 2 min.

### Reduced graphene oxide

The rGO was synthesized starting from graphite following a modified Hummers method and a subsequent chemical reduction [55]. To cover the substrates with a uniform layer of reduced graphene oxide 200 μL of a 0.25 μg·mL solution containing 1:1 (v/v) water and isopropanol was deposited in the middle on the surface and allowed to settle for 5 min. The solvent with excess on graphene was removed by spin coating (Laurell Spin Coater WS-400-6NPP-LITE; Laurell Technologies Corporation, North Wales, Pennsylvania, USA) at 1000 rpm for 11 min and 2500 rpm for 1 min. After treatment the slides were rinsed with ethanol and dried with N_2_.

### Surface plasmon resonance spectroscopy

The SPR analysis was performed with a BioSuplar SPR instrument (Mivitec GmbH, Sinzing, Germany) using a F1-65 glass prism installed on a swivel carriage. The substrate is placed on the top face with index-matching fluid between the chip and the prism. A flow cell with two channels is placed on the chip and samples were passed through the cell. The device operates with a laser illumination at 650 nm. The bulk sensitivity to refractive index (intensity per refractive index units (RIU)) was measured with aqueous sucrose solutions (1–8% w/w) covering a range of 1.33–1.35 RIU. For measurements the change in intensity of reflected light at a fixed angle was monitored. SPR slides covered with a continuous gold film of 45 nm thickness were obtained from Mivitec GmbH. Four environment water samples were taken from Lake Starnberg (Starnberg, Germany), Ionian Sea (Laganas, Greek), Lake Garda (Limone sul Garda, Italy) and River Danube (Regensburg, Germany) in sealed glass bottles and stored in the dark until measurement. No sample pre-treatment was applied. Interaction of the respective DEP solution was allowed for 6 min. To remove unbound DEP and ensure an adsorption-based signal change on the sensor surface, a 10 min washing step was performed after each DEP solution analysis.

### Raman microscopy measurements

Raman microscopy measurements (DXR Raman microscope, Thermo Fisher Scientific GmbH, Dreieich, Germany) were performed at 532 nm laser excitation (10 mW) and with a 50 μm slit. The spectra were acquired for 1 s and averaged over ten measurements. The microscopic image and the Raman maps were taken at 100 times magnification with a MPlan N objective (100×/0.90 BD, Olympus SE & Co. KG, Hamburg, Germany).

## Supporting Information

Figure S1: Respective size distribution analysis of the particles; Figure S2: Time dependence of the particle diameter reduction; Table S3: Fitting parameter for the interaction of DEP with rGO on various substrates.

File 1 Additional experimental data.
