# Supplementary material for: Graphene-enhanced plasmonic nanohole arrays for environmental sensing in aqueous samples
Source: Beilstein J Nanotechnol. 2016 Nov 1;7:1564–73. doi: 10.3762/bjnano.7.150 (PMC5238696; doi:10.3762/bjnano.7.150)
Supplement: File 1 — Additional experimental data. [file Beilstein_J_Nanotechnol-07-1564-s001.pdf]

Supporting Information

for

Graphene-enhanced plasmonic nanohole arrays for environmental sensing in aqueous samples

Christa Genslein[1], Peter Hausler[2], Eva-Maria Kirchner[1], Rudolf Bierl[2], Antje J. Baeumner[1] and Thomas Hirsch*[1]

Address: [1]Institute of Analytical Chemistry, Chemo and Biosensors, University of Regensburg, 93040 Regensburg, Germany and [2]Sensorik-ApplikationsZentrum, OTH Regensburg, Franz-Mayer-Str. 1, 93053 Regensburg, Germany

Email: Thomas Hirsch* - thomas.hirsch@ur.de

* Corresponding author

Additional experimental data

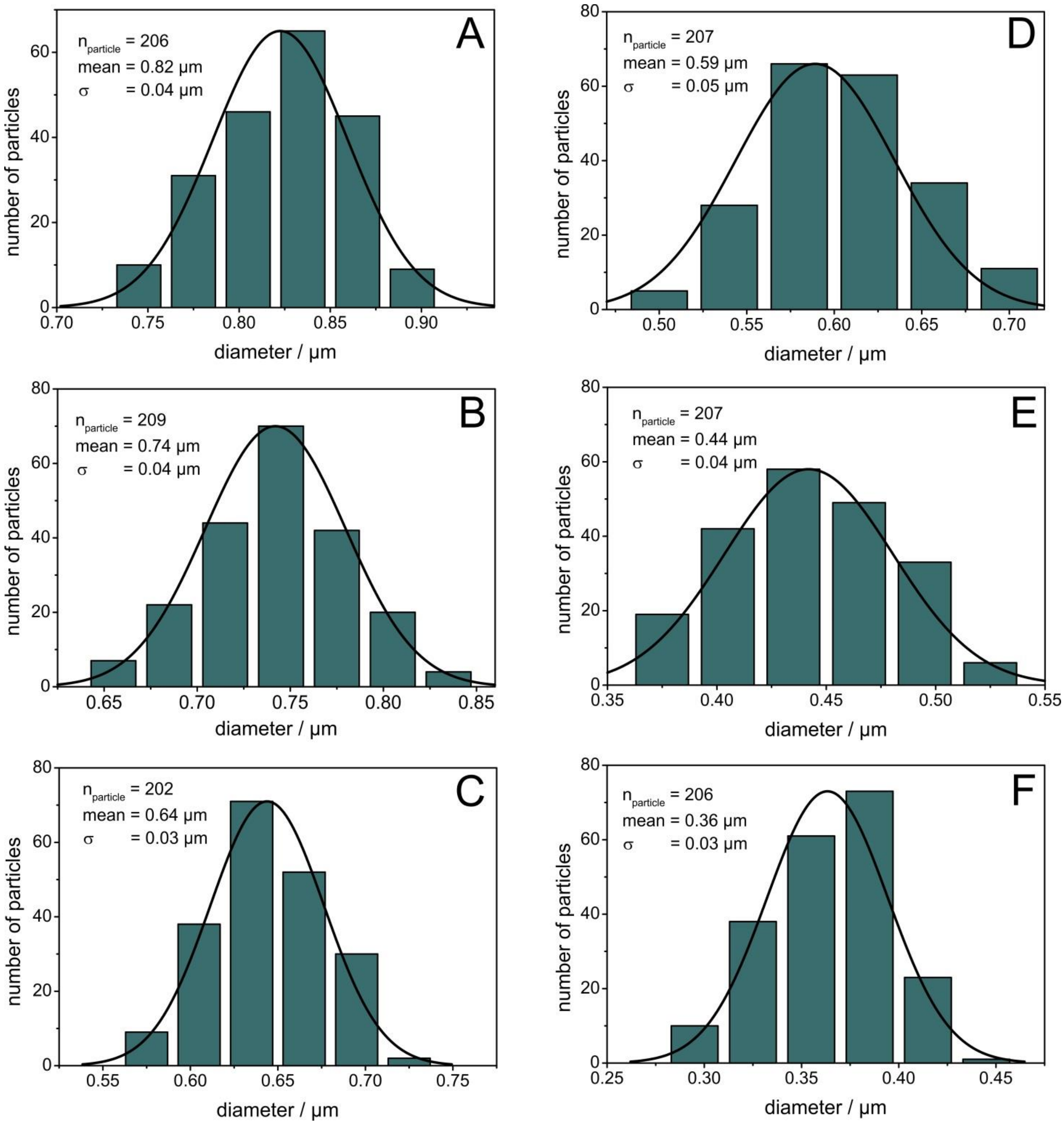

Figure S1: The respective size distribution analysis of the particles is from the SEM images (compare Figure 3). A Gaussian function was used for fitting. Substrates were covered by ~45 nm Au with a ~3 nm Ti adhesion layer. A decrease in the diameter of the polystyrene particles with an increase of the etching time from 8 min (A) to 28 min (F) can be seen.

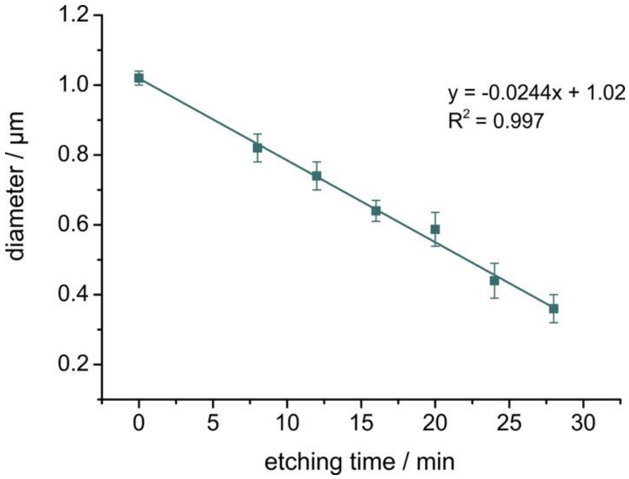

Figure S2: Time dependence of the particle diameter reduction. Particles have a starting diameter of 1.02 µm. Etching is performed using reactive ion etching with oxygen plasma. Standard deviation is taken from the respective size distribution of the SEM image analysis (Figure S1).

Table S3: Fitting parameter for the interaction of DEP with rGO on various substrates. The Langmuir model was used.

substrate		*K* / µM	K_A / µM^{-1}	R^2
continuous film		0.17 ± 0.03	6 ± 1	0.9693
nanohole array with *D*/*P*	0.35	0.22 ± 0.05	5 ± 1	0.9149
	0.43	0.15 ± 0.02	7 ± 0.9	0.9847
	0.58	0.20 ± 0.04	5 ± 1	0.9585

$$\Delta s = \frac{c}{K + c},$$

where Δ*s* is the signal change, *c* is the DEP concentration and *K* represents the equilibrium dissociation constant.
